# Resection arthrodesis for giant cell tumors around the knee

**DOI:** 10.4103/0019-5413.32043

**Published:** 2007

**Authors:** Sudhir K Kapoor, Akshay Tiwari

**Affiliations:** Department of Orthopaedics, Lady Hardinge Medical College and Associated Hospitals, New Delhi - 110 001, India

**Keywords:** Giant cell tumor; knee, resection arthrodesis

## Abstract

**Background:**

Giant cell tumors (GCTs) of bone are aggressive benign tumors. Wide resection is reserved for a small subset of patients with biologically more aggressive, recurrent and extensive tumors. As the patients affected with GCT are young or middle-aged adults with a normal life expectancy, arthrodesis is an attractive option for reconstruction in these patients.

**Materials and Methods:**

Thirty-six patients of mean age 33.1 years with Campanacci Grade III giant cell tumors around the knee (20 distal femoral and 16 proximal tibial) were treated with wide resection and arthrodesis from January 1996 through January 2006. Arthrodesis was performed using plating with free fibular graft (n = 18), IM nail with free fibular graft (n = 8) and IM nail combined with ring fixator using bone transport (n = 10).

**Results:**

Fusion after the first surgery was achieved in 77.7%, 75% and 90% of the patients in the three groups respectively. Local recurrence was seen in two patients and repeat surgery for nonunion/ graft fracture had to be done in four patients and two patients in the plating and nailing groups respectively.

**Conclusion:**

Wide resection and arthrodesis in aggressive GCTs around the knee is a good treatment option. IM nail combined with a ring fixator seems to be a good method of arthrodesis with high fusion rates, least shortening and early rehabilitation.

Giant cell tumor (GCT) of bone is a benign aggressive tumor of bone.^1^ The reported incidence, however, is greater in the east as compared to the west.^2^,^3^ The principal treatment modality of this locally aggressive benign tumor is surgery, radiotherapy being reserved for the inaccessible tumors.^4^ The adequate removal of the tumor does minimize the risk of recurrence.^5^ However, the aggressive resection must be weighed against the fact that GCTs are benign tumors.^6^ The surgical treatment of this tumor has always been controversial, with the desired treatment being a balance between adequate removal and retention of function.^7^ The available surgical options include curettage with bone grafting, extended curettage using chemical cauterization/ cryosurgery with bone grafting, cementation with or without delayed bone grafting and wide resection with suitable reconstruction. While extended curettage has produced good results in well-contained GCTs, it has not done so in tumors with cortical breach and large soft tissue masses. Hence, wide resection is reserved for a small subset of patients with biologically more aggressive, recurrent and extensive tumors.^4^ The knee is the commonest region affected by GCT^1^ and the reconstruction options after a resection of such a large tumor around the knee include allograft and suitable stabilization, custom-made hinged knee prostheses and arthrodesis. The use of allografts are restricted by the nonavailability of facilities of patient-matched allografts in India; hinged knee prostheses have the disadvantage of requiring multiple revision surgeries. The patients affected with GCT are young or middle-aged adults with a normal life expectancy and arthrodesis is an attractive treatment option for these patients. We report the outcome in 36 patients with aggressive GCTs of the lower end of femur or upper end of tibia, treated with wide resection and arthrodesis of the knee over a period of 10 years.

## MATERIALS AND METHODS

**Figure 1:**
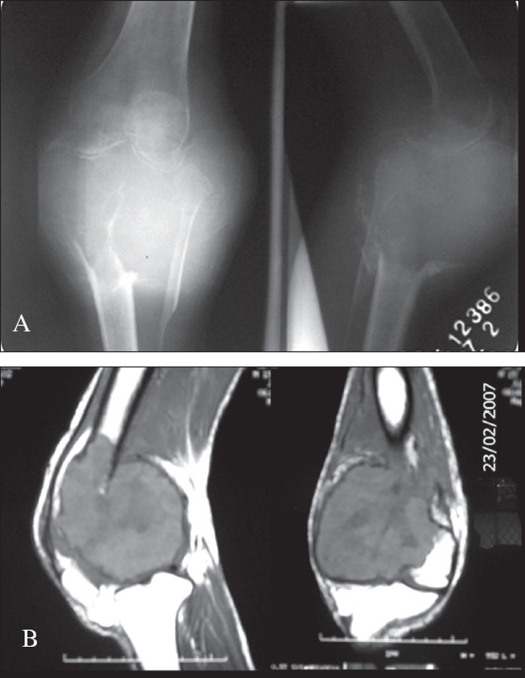
X-ray showing involvement of proximal tibia A) and MRI showing distal femoral involvement B) with tumors with gross cortical destruction and large soft tissue component (in two different patients)

Forty patients of giant cell tumors around the knee were treated with resection and arthrodesis from January 1996 through January 2006. Four cases had a follow-up of less than two years hence the results of 36 patients are reported here. Three groups of patients consisting 18, 8 and 10 cases were treated respectively by arthrodesis using plating and free fibular grafting, IM nailing alone with free fibular grafting and IM nailing combined with bone transport using ring fixator. There were 19 female and 17 male patients, with the mean age of 33.1 years (range 23 to 45 years). There were 27 primary and nine recurrent cases and all the tumors were Campanacci Grade III tumors at presentation [Figure 1]. Of the nine cases with recurrence, five had been treated at other institutes with curettage and bone grafting, while the other four had been treated by us with extended curettage (three using high speed burr, absolute alcohol and bone grafting and the fourth using cementation). The time to recurrence was an average of 1.3 years after the primary surgery.

The tumor was located in the distal femur in 20 cases and proximal tibia in 16 cases. None of the tumors had a neurovascular deficit. The radiographic findings were typical in all the cases; an eccentric or widely destructive (in late cases) geographic lesion without a sclerotic margin, epiphysiometaphyseal in location, with destruction of one or more cortices and a large soft tissue mass [Figure 1].

The average tumor size on plain radiographs was 13.5 cm × 8.5 cm. MRI could be obtained in 26 patients. (it was not available in the earlier cases), showed the extent of the soft tissue component, its relation with the neurovascular bundle, breach of the articular surface and the intramedullary extent of the disease. Thus, MRI was helpful in determining the feasibility of limb salvage, in knowing in advance the displacement of the neurovascular bundle if any and in planning a more precise resection length. All the cases were subjected to a biopsy (25 open biopsy and 11 core biopsies) and were taken up for surgery on the next available OT date.

A wide resection was performed in all the patients using the medial parapatellar and subvastus incisions for lower femoral GCTs and the same incision extended distally for the upper tibial tumors. The incision, however, had to be modified in six cases, because of the previous biopsy/ surgery done at other hospitals. Out of a total of 16 cases of upper tibial resections, six cases required removal of proximal fibula due to involvement / close proximity of the tumor to proximal tibiofibular joint. In all these cases, the anterior tibial artery was routinely ligated to aid in isolation of the neurovascular bundle.The medial head of gastrocnimeus was routinely transferred anteriorly over the fusion site in all the cases of upper tibial resections.^8^ The mean resection length was 14.4 cm and the intended shortening was 2.5 cm.

**Figure 2:**
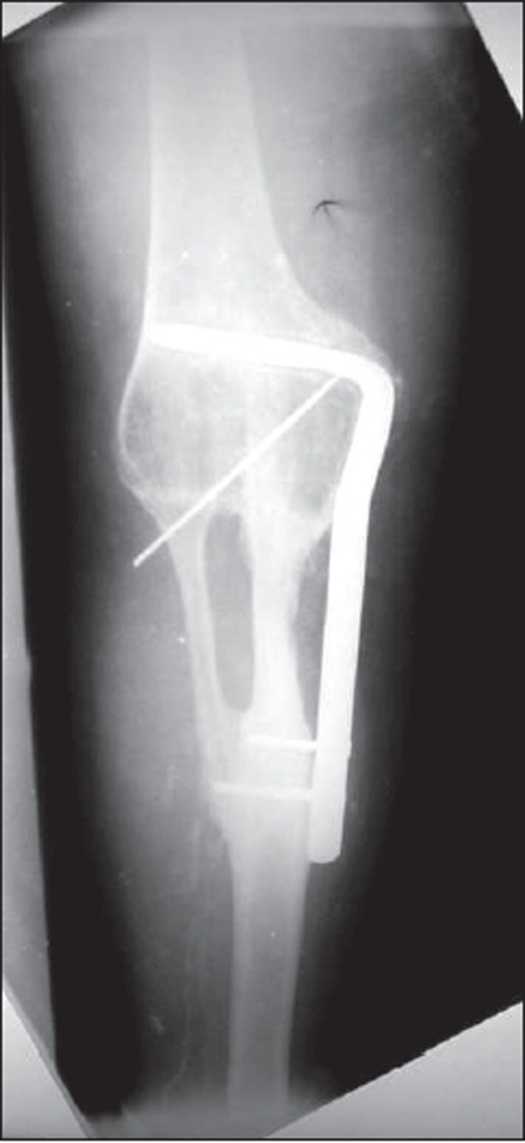
3 years followup after resection arthrodesis with blade plateshowing union and no recurrence

The initial 18 consecutive cases (10 femoral and eight tibial) were treated with resection and arthrodesis by autogenous fibular graft and plating. A 4.5 mm blade plate with a minimum of four and preferably eight cortices was applied on the lateral aspect in neutralization mode, with dual fibular autografts spanning the defect created by the resection (one of the grafts lodged in the medullary canal on either side) [Figure 2]. The patients were kept non-weight-bearing with a high above-knee cast for 20-24 weeks, with gradually increasing partial weight-bearing using a cylinder cast thereafter. The cast was removed at an average of 40-44 weeks and the patients could return to vigorous work after an average of 66-70 weeks. The average time to fusion was 60 weeks. All the patients were mobilized on an above-knee brace.

**Figure 3:**
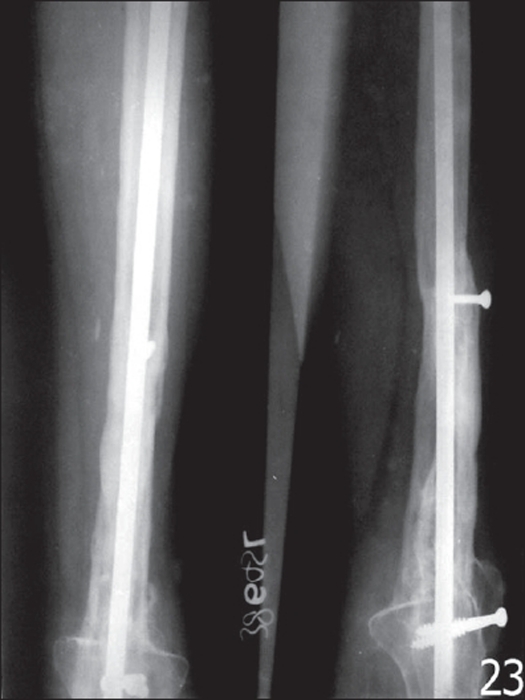
Resection arthrodesis with IM nail showing solid union (3.5 years postoperative)

Arthrodesis with long IM nail and fibular autograft was done in the next eight consecutive cases of lower femoral (n=4) and upper tibial (n=4) tumors. A long IM nail was placed in a retrograde fashion, extending from the greater trochanter to distal tibial metaphysis. Dual free fibular grafts were placed spanning the resection length [Figure 3]. The patients were allowed partial weight-bearing with a long leg cast by about 28 weeks postoperatively. The patients however, returned to vigorous activity at an average of 52-56 weeks. The average time to fusion was 52 weeks.

**Figure 4:**
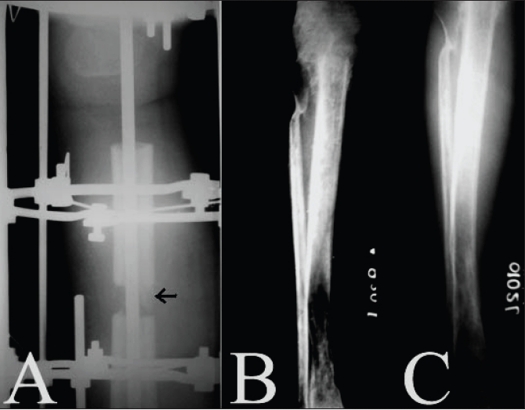
A) X-ray after resection of GCT of pxoximal tibia and distal tibial corticotomy. The tibia has been fixed with an intramedulary nail and ilizarov external fixator. Arrow shows regenerate at the corticotmy site. B) Follow up X-ray showing complete docking at the resection site and regenerate at the corticotomy site. C) X-ray shows union at the resection site and consolidation at the corticotomy site.

A total of 10 cases (six femoral and four tibial) underwent arthrodesis by long intramedullary nail combined with ring fixator. We used a long IM nail of a small diameter to stabilize the limb after resection of the tumor and a ring fixator was applied thereafter. Corticotomies were performed at femoral or tibial side and a bone transport was done [Figure 4].

In the case of two upper tibial tumors, however, the technique was modified slightly. The upper end of the fibula was fixed to the femur with the help of a K wire. A femoral osteotomy allowed bone transport and maintenance of limb length and a free fibular graft was placed in a second stage when radiological union was evident between the fibula and the femur. Ring fixator in combination with IM nail being a very stable construct, partial weight-bearing could be allowed at two to three weeks in most of the cases. The fixator was removed when the required regenerate consolidated and the patient was mobilized with an above-knee brace by the end of the first year. The average time to fusion was 44 weeks. Mean lengthening achieved was 11.2 cm (13.9 cm if the two tibial tumors with femorofibular arthrodesis are excluded).

## RESULTS

At an average follow-up of 3.35 years (range 2-8 years), none of the patients showed metastasis or malignant transformation. The outcome of the patients at final follow-up is showed in Table 1.

**Table 1:** Outcome of resection arthrodesis in 36 patients with giant cell tumors around the knee

Modality of Treatment	No. of patients	Average follow-up (years)	Average tumor size (cm)	Fusion (1^st^ surgery) %	Complications			Average shortening (cm)
						
					Deep infection requiring amputation	Wound dehiscence	Fracture of graft	
Plating	18	3.7	14.5	77.7	1	4	4	3
Nailing	8	3.4	13	75	None	None	None	4.5
Ring fixator	10	2.75	15.5	90	1	None	None	2.5

Out of a total of 18 patients treated with plating, fusion was achieved in 14 (77.7%). Two patients had to be subjected to amputation-one case developed necrosis of skin flap, deep infection and exposure of the graft, while the other developed local recurrence. In two other patients, a nonunion of atrophic type was seen at the graft-host interface. Successful fusion was seen in both these patients following autogenous bone grafting with above-knee cast. Late fracture of the fibular graft was seen in four patients, all the fractures occurring at the middle third of the fibular autograft. The fractures were seen 28 weeks, 40 weeks, 46 weeks and 50 weeks following the surgery. All the fractures were united with cortico-cancellous bone grafting. 4 patients had full thickness flap necrosis exposing bone and / or implant while 2 patients had only skin or subcutaneous tissue necrosis. The postoperative wound dehiscence was seen in four upper tibial resections. One out of these four patients required a myocutaneous flap. One patient died four years after surgery due to an unrelated cause (chronic liver disease).

**Figure 5:**
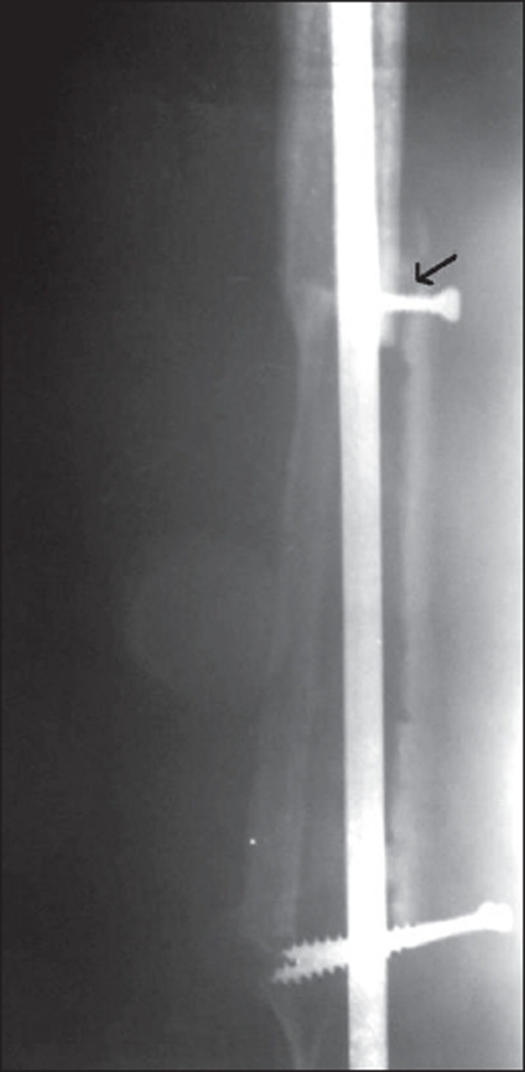
Resection arthrodesis using IM nail shows nonunion of graft at 1.5 years (arrow)

Six (75%) out of eight cases treated by arthrodesis using IM nail achieved fusion, while two cases had unsatisfactory radiographic union and required bone grafting [Figure 5]. One patient interestingly developed a local soft tissue recurrence four years after the initial surgery. The patient remains disease-free till date, after he was operated for the local recurrence. All the patients were free of disease at final follow-up. No patient had early or late fracture of graft and none had wound dehiscence.

Nine of the patients treated by ring fixator showed good consolidation of the regenerate and achieved successful fusion (an average of 55 days was required to achieve the desired lengthening), while one patient developed wound dehiscence with deep intramedullary infection. The infection did not resolve with removal of intramedullary nail and debridement and had to be subjected to amputation. None of the patients developed a fracture of the graft. Two patients developed an equinovarus deformity of the ankle secondary to bone transport, both of which could be managed conservatively. None of the patients developed transient or permanent nerve palsy. Superficial pin site infection at one or more sites, at some stage of follow-up was seen in all patients. It was mostly controlled by antiseptic dressing and appropriate oral antibiotics, except in two patients who required change of wires.

## DISCUSSION

Treatment of GCTs has been restricted mostly to extended curettage and cementing or bone grafting.^9^ However, for the aggressive forms with significant soft tissue extension, intralesional surgery is fraught with a greater risk of recurrence. While the functional outcome of patients undergoing wide resection is not as good as those with intralesional surgery, wide resection is indicated in patients with aggressive GCTs with large soft tissue extension and those with recurrence.^4^,^10^,^11^

Resection arthrodesis of the knee has been found to be a useful procedure for tumors around the knee.^12^–^15^ While reconstruction with prostheses and allografts^16^ provides better function to the limb in the short term by restoring the joint motion, they may not be an ideal choice for young vigorous adults with a normal life expectancy. These patients are likely to outlive the expected life of a hinged prosthesis even after one or more revisions. Further, the use of free autologous grafts is not subject to the unpredictability of union of the grafts brought about by radiotherapy and chemotherapy. Thus, an arthrodesis is the natural choice for these patients.

The knee is the commonest joint having GCT in its vicinity, accounting for nearly 50% of all cases.^11^ It is a weight-bearing joint with great stresses on the arthrodesis site, hence making it a difficult joint to fuse. While knee arthrodesis generally gives good results with the various techniques evolved over time, it is associated with a higher complication rate in patients who have undergone resection for tumors with considerable gaps including nonunion, breakage of grafts, infection and late fractures. Moreover, the salvage of such limbs with failed arthrodesis is all the more difficult, many ending up with amputation.

Similar rates of union (92%) following resection arthrodesis of the knee in various tumors have been reported by other workers.^13^,^15^ They used a hemicortical graft from the available bone (femur or tibia in cases with tibial and femoral tumors respectively). A good functional outcome has been reported by these workers following knee arthrodesis in these cases and they recommend the procedure as an alternative to prostheses in all malignant and potentially malignant tumors.

We have used three different methods over the last 10 years for arthrodesis of the knee undergoing resection for GCT. The method used in earlier cases was plating with autogenous fibular grafting. We find this method technically easier but it had significant occurrence of complications. The profile of the implant makes soft tissue coverage difficult. Of particular concern was the impingement of the corner of the plate under the skin which broke down at times. Moreover, the incidence of fractures of the graft was higher in the plating group, probably because of the stress shielding provided by the plate, it being a load-bearing implant (as opposed to a load-sharing implant such as a nail). Also, biomechanically, the plate as an implant is inferior to the intramedullary nail, being some distance away from the line of direct load (the medullary canal).^17^ Hence, plate osteosynthesis should probably be combined with a vascularized fibular graft rather than a free fibula, for earlier graft uptake and hypertrophy.^18^

Long IM nail with fibular autograft has been used with success for a long time. It is a one-time method, technically easy and coverage of implant is never a problem. However, this group had a high incidence of nonunion at graft-host interface in our series. As the medullary canal was occupied by the nail itself, the graft could not be placed in the medullary canal (on the other hand, at least one of the grafts was always placed in the medullary canal when plating was used for fixation). This could explain a higher incidence of nonunion in the nailing group in our series.

Arthrodesis with a long IM nail and ring fixator is the latest of the methods we have used and seems to be a promising technique.^14^ The rate of nonunion was low with this technique and was found to be much less cumbersome than the long leg cast once the fixator was reduced in bulk by removing the proximal and the distal most rings. The patients could join work before the end of the first year, rate of late fractures was low (it may be partly because of a shorter period of follow-up as compared to the other two groups) and the length of the limb could be restored to as near the normal as desired. A nail of a small diameter allows bone transport without deformities, while preserving the endosteal blood supply.^14^ However, the ring fixator has inherent problems such as recurrent pin tract infections and deformities of the ankle and foot (mostly equinovarus; knee deformities are avoided because of the intramedullary nail). The patient should be intelligent enough to comply with the distraction regime and take care of the pin sites.

## CONCLUSION

Resection arthrodesis of the knee is a good treatment option in patients with GCTs around the knee, once wide resection becomes mandatory. The intramedullary nail combined with ring fixator has been better than the other two techniques, with high fusion rates, early rehabilitation and good control over the ultimate limb length.
